# A dynamic causal modeling of the second outbreak of COVID-19 in Italy

**DOI:** 10.1007/s10182-023-00469-9

**Published:** 2023-02-07

**Authors:** Massimo Bilancia, Domenico Vitale, Fabio Manca, Paola Perchinunno, Luigi Santacroce

**Affiliations:** 1 Department of Precision and Regenerative Medicine and Ionian Area (DiMePRe-J), University of Bari Aldo Moro, Policlinic University Hospital – Piazza G. Cesare 11, 70124 Bari, Italy; 2 MEMOTEF Department, University of Roma La Sapienza, Via del Castro Laurenziano 9, 00161 Rome, Italy; 3 Department of Education, Psychology, Communication (ForPsiCom), University of Bari Aldo Moro, Palazzo Chiaia Napolitano – Via S. Crisanzio 42, 70122 Bari, Italy; 4 Department of Business and Law Studies (DEMDI), University of Bari Aldo Moro, Largo Abbazia di Santa Scolastica 53, 70124 Bari, Italy; 5 Department of Interdisciplinary Medicine (DIM) and Microbiology and Virology Unit, University of Bari Aldo Moro, Policlinic University Hospital – Piazza G. Cesare 11, 70124 Bari, Italy

**Keywords:** COVID-19, Causal analysis, Gaussian processes, State-space models, Bayesian modeling, Bayesian computations

## Abstract

While the vaccination campaign against COVID-19 is having its positive impact, we retrospectively analyze the causal impact of some decisions made by the Italian government on the second outbreak of the SARS-CoV-2 pandemic in Italy, when no vaccine was available. First, we analyze the causal impact of reopenings after the first lockdown in 2020. In addition, we also analyze the impact of reopening schools in September 2020. Our results provide an unprecedented opportunity to evaluate the causal relationship between the relaxation of restrictions and the transmission in the community of a highly contagious respiratory virus that causes severe illness in the absence of prophylactic vaccination programs. We present a purely data-analytic approach based on a Bayesian methodology and discuss possible interpretations of the results obtained and implications for policy makers.

## Introduction

Until recently, numerous papers have dealt with the dynamics of the first COVID-19 outbreak (e.g., Gnanvi et al. 2021). Most of them measured the impact of national containment measures and other non-pharmaceutical interventions (NPIs) on the reproductive number $R_t$ (the average number of new infections that each infected case generates during the course of infection; Gostic et al. 2020). In the absence of a specific vaccine, NPIs were the only possible intervention until at least the end of 2020.

However, the analysis of the second outbreak and the search for the possible causes of its large amplitude is an equally interesting problem with significant practical implications. The contagion dynamics that Italy experienced between the end of the lockdown and the beginning of the second wave was observed in all European countries: a gradual relaxation of restrictions and a slow resurgence of new cases of infection in early July 2020. We can consider the implementation of restrictive measures and their relaxation as the application of two forces with opposite directions on the contagion mass. The lack of literature on the latter aspect is most likely due to some technical difficulties, in particular the fact that $R_t$ is a sample estimate that is subject to large uncertainty when the incidence is low (Cori et al. 2013), as is the case just in the period between July and October 2020 that precedes the onset of the second outbreak.

Because of these difficulties, we chose a different perspective to analyze the spread of COVID-19 disease in the period between the end of the first lockdown and the beginning of the second wave. First, this study is a single-country study focusing on Italy. Some governmental decisions (and the resulting decisions of the Regions) implemented between June and September 2020 triggered numerous controversies about their cause–effect relationship with the second outbreak, without any resolution. We focused specifically on the two most debated interventions: first, the DPCM (Decreto del Presidente del Consiglio dei Ministri = Decree of the Prime Minister) of June 11, 2020, which provided for a significant relaxation of restrictions. This decree was followed by other accompanying measures enacted by most Regions, which also liberalized summer vacations throughout the national territory. This new freedom of movement was often blamed as the cause of the resurgence of contagion after the first outbreak. The second measure is the reopening of schools, which occurred in most Regions on September 14, 2020. The uncertainty about the actual impact of these important decisions is worthy of further exploration because it provides an unprecedented quasi-experimental framework for evaluating the causal relationship between policy actions and the spread of severe and highly contagious respiratory disease in the absence of a vaccination program. The approach we use is based on a counterfactual analysis (Höfler 2005; Rubin 2011; Morgan and Winship 2014) and a Bayesian methodology for estimating state-space dynamic time series models. The method used has the fundamental advantage that it does not require the estimation of $R_t$.

The paper is organized as follows. Section 2 describes the data and a smoothing procedure based on Gaussian processes to remove a periodic component due to variability in swabs administered on different days of the week. Section 3 is devoted to the presentation of the Bayesian methods used to dynamically estimate the causal impact of the interventions on the spread of COVID-19 infection and its relationship to the onset of the second outbreak. The results are presented in detail in Sect. 4, both at national and regional levels. Finally, Sect. 5 attempts to provide an explanation for the results obtained and Sect. 6 draws conclusions.

## Data and preprocessing

### Data sources

Outcome data at the national level consist of the time series of the daily total number of confirmed cases in Italy from February 21, 2020, when the first cases were officially reported, to January 14, 2021. Scraping of incidence data was performed directly from Wikipedia, using the template *COVID-19 pandemic data/Italy medical case* (Wikipedia 2021), which summarizes daily data from Regions and Autonomous Provinces (PP/AA) collected by the Italian Civil Protection. Specifically, we used the table *Daily COVID-19 cases in Italy by Region*, which is updated daily and contains data on the number of new cases, deaths, and admissions to intensive care units, as well as the number of RT-PCR tests performed daily. Scraping was performed in R 4.1.2 using the library rvest (R Core Team 2021; Wickham 2021). The data were further minimally preprocessed to: (1) extract the parts of the table that were of immediate interest; (2) remove some unwanted non-printable characters that were present in the original HTML code; (3) extract the daily number of new cases and swabs; and (4) create a data structure that could be used in R for subsequent data analysis.

**Fig. 1:**
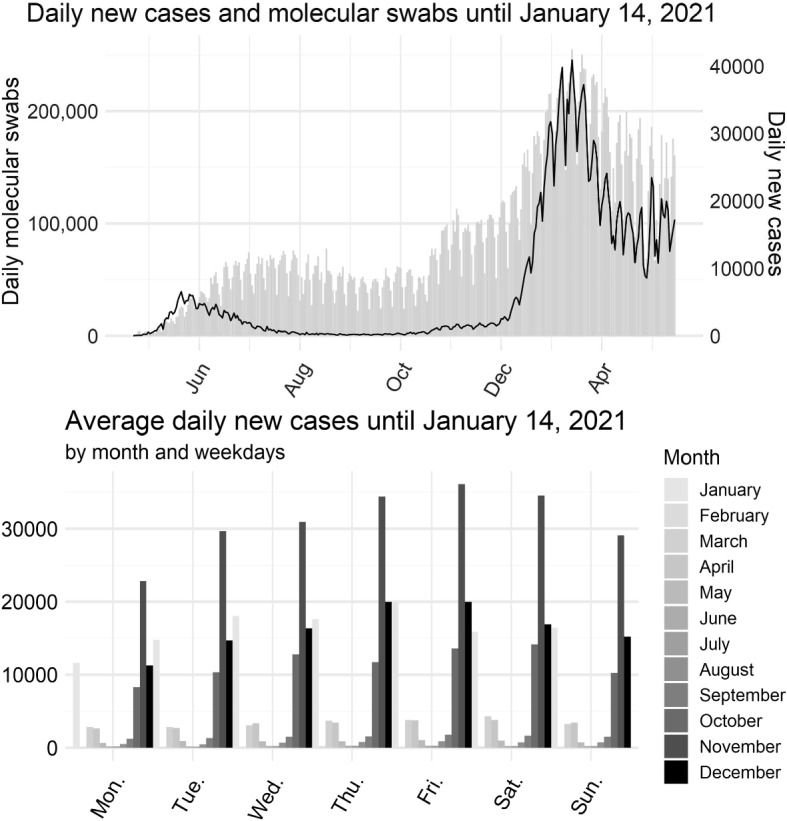
(Top) Number of daily new cases diagnosed and RT-PCR molecular tests performed in Italy between February 21, 2020, and January 14, 2021. Daily new cases are represented by the solid black line; the light gray bar graph shows the number of molecular swabs processed daily. (Bottom) Average number of new cases diagnosed daily in Italy between February 21, 2020, and January 14, 2021, stratified by month and day of the week

Average daily temperatures for regional capitals were taken from the ILMETEO.it historical archive (Il Meteo 2021), except for the data for Aosta (regional capital of Valle d’Aosta), which were available on the Regione Valle d’Aosta meteorological data portal (Regione Valle D’Aosta 2021). The end of the observation window was set to January 14, 2021, because from January 15, 2021, the counting method was changed to include SARS-CoV-2 antigen rapid tests.

The graphical representation of the data can be found in Fig. 1 (top panel). The curve of daily new cases shows an irregular, wavelike pattern as the ratio of the number of new cases to the number of subjects tested is locally constant (in the sense that it is approximately constant over short time windows), whereas the number of new cases is systematically lower on some days of the week as fewer tests are performed. (An example of this pattern is seen on Monday when confirmed new cases are largely tested on the previous weekend.) This periodic component is even more evident in the bottom panel of Fig. 1. There we can see how the average number of newly diagnosed cases decreases on weekends and Mondays due to the aforementioned decrease in the number of tests administered.

### Preprocessing: smoothing with Gaussian processes

The cyclic pattern present in the data takes the form of a weekly oscillation with time-dependent amplitudes. This oscillation exhibits phase coupling with the daily number of tests processed, in the sense that the phase difference between the series of daily new cases and the series of daily swabs remains constant for obvious reasons. The presence of such a non-stationary waveform in the data must be considered a perturbation that does not reflect the actual dynamics of contagion. Rather, it is the result of variability in human activities, and its effects can only be quantified if different scenarios are considered in the data analysis. For this reason, we preliminarily created an alternative dataset in which the periodic component was filtered out by an algorithm based on Gaussian processes (Zhang et al. 2011). In the proposed approach, the trajectory of daily counts was decomposed into the sum of four components:

➀ A smooth component describing long-term variation (trend).
➁ A component describing faster, short-term variation.
➂ A non-stationary periodic component with period $s = 7$ and time-dependent amplitudes, describing the cyclic pattern present in the data.
➃ The noise, i.e., the unstructured residuals not explained by the other components.

More formally, we have the following additive model for the common input variable $t\in \mathbb R$ (time index):

1 $$\begin{aligned} f(t)=f_1 (t)+f_2 (t)+f_3 (t)+\varepsilon _t, \quad t=1,2,\ldots ,n, \end{aligned}$$

where $\varepsilon _t \sim \mathcal N(0,\sigma ^2_\varepsilon )$ is a realization from a Gaussian white noise process. Assuming that the components of this process are mutually independent, the total covariance function (or kernel) $\kappa (t, t')$ describing the correlation structure between neighboring points is given by:

2 $$\begin{aligned} \kappa (t,t')=\kappa _1(t,t')+\kappa _2(t,t')+\kappa _3(t,t') + \sigma ^2_\varepsilon \mathbbm {1}(t=t'), \end{aligned}$$

where the different terms represent the different scales and periodicities in the data. Specifically:

- $f_1(t)$ is a random Gaussian function modeling smooth long-run variation ➀, using a squared exponential kernel:
  3 $$\begin{aligned} \kappa _1(t,t') = \sigma _1^2\exp \left( - \frac{(t - t')^2}{2\ell ^2_1} \right) , \end{aligned}$$
  where the amplitude $\sigma _1^2$ determines the maximum excursion of the trajectories, and the parameter $\ell _1$ controls the degree of smoothing. It can be shown that the expected value of the number of crossings of a trajectory with the *x*-axis in the unit interval is equal to $(2 \pi \ell _1)^{-1}$. Thus, as $\ell _1$ increases, the function becomes less irregular and smoother as it wiggles less rapidly above and below the *x*-axis.
- $f_2(t)$ models the short-term variation ➁ using a squared exponential kernel with different parameters:
  4 $$\begin{aligned} \kappa _2(t,t') = \sigma _2^2\exp \left( - \frac{(t - t')^2}{2\ell ^2_2} \right) . \end{aligned}$$
- $f_3(t)$ represents the non-stationary waveform with time-dependent amplitudes ➂. In this case, the correlation structure can be conveniently described by a locally periodic kernel with period $s=7$:
  5 $$\begin{aligned} \kappa _3(t,t') = \sigma _3^2\exp \left( - \frac{(t - t')^2}{2\ell ^2_{3.1}} \right) \exp \left( - \frac{2\sin ^2 \frac{\pi (t - t')}{s}}{\ell ^2_{3.2}} \right) . \end{aligned}$$

**Fig. 2:**
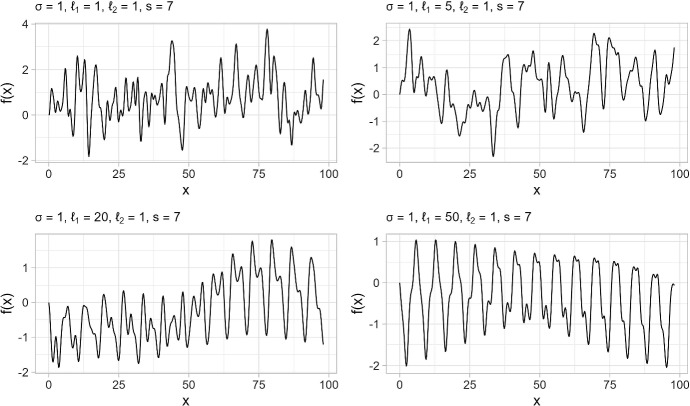
Four trajectories (discretized over $m = 500$ points) from a Gaussian process with locally periodic covariance function of period $s=7$, for different values of $\ell _1$ and $\ell _2$

While from a Bayesian perspective there are several ways to evaluate the goodness of fit of the model and its predictive performance, as well as to select a model from a set of candidate models (Vehtari and Ojanen 2012; Gelman et al. 2014), our approach is quite informal in the sense that the proposed specification is based on the structural knowledge of the domain of study. For example, the locally periodic kernel $\kappa _3(t,t')$ is used to model the irregular waveform in the observed daily counts. Figure 2 shows some synthetic trajectories of a Gaussian process with kernel $\kappa _3$ for selected values of the parameters. These trajectories are periodic time series where the cyclic pattern is non-stationary and has no well-defined amplitude.

The specification of the prior distributions over the model parameters is as follows:

6 $$\begin{aligned} \sigma _1,\sigma _2,\sigma _3&\overset{\mathsf {ind.}}{\sim }\text {Half-}\mathcal {N}(0,1), \end{aligned}$$

7 $$\begin{aligned} \ell _1,\ell _2,\ell _{3.1},\ell _{3.2}&\overset{\mathsf {ind.}}{\sim } \text { Inv-Gamma }(5,5). \end{aligned}$$

This joint prior specification is a generic weakly informative choice that attempts to introduce weak prior assumptions about the true location of the unknown parameters into our analysis (Gelman 2006). The $\text {Half-}\mathcal {N}(0,1)$ indicates the distribution of the absolute value of a random variable whose distribution is $\mathcal N(0,1)$. The $\text {Inv-Gamma}(\alpha ,\beta )$ with shape parameter $\alpha > 0$ and scale parameter $\beta > 0$ is the distribution of the inverse of a random variable with distribution $\text {Gamma}(\alpha , \beta )$. With $\alpha = \beta = 5$, we have a prior distribution approximately centered around 1 (the expected value is $5/4 = 1.25$) and quite diffuse, since the interval between the quantiles 0.025 and 0.975 is (0.49, 3.08).

It is also important to note that there is a fundamental ambiguity in which parameters are associated with each component of the Gaussian process, since the likelihood of the sum is invariant to each permutation of the indices. With a weakly informative prior, the posterior distribution inherits the permutation invariance. One way to resolve this ambiguity is to restrict the study of posterior modes to a single order of parameters, a trick that can be interpreted as a way to make an exchangeable prior non-exchangeable. It can be proved that the resulting inferences are correct after imposing an artificial identifiability constraint (Betancourt 2017). Moreover, this approach can often separate a set of symmetric posterior modes (Papastamoulis 2016). The ordering we used for our problem naturally reflects the relative weight of the additive components of the Gaussian process:

8 $$\begin{aligned} \pi (\sigma _1,\sigma _2,\sigma _3)\mathbb I \left( \sigma _2<\sigma _3 <\sigma _1 \right) . \end{aligned}$$

**Fig. 3:**
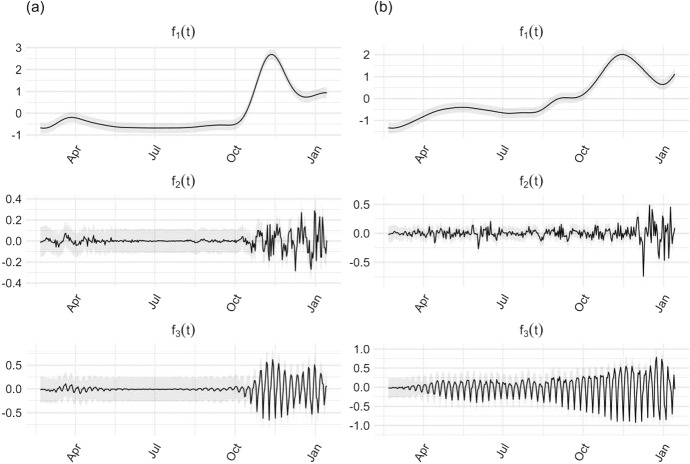
Decomposition of daily number of new cases (**a**) and daily number of molecular swabs administered (**b**) into three linearly superimposed components estimated using a sum of Gaussian processes. The proposed decomposition model includes: (1) Long-term smooth trend $f_1$. (2) Short-term fluctuation $f_2$. (3) Non-stationary weekly periodic component with time-dependent amplitudes $f_3$. (4) Noise (not shown here). The gray-shaded areas represent the approximate 95% posterior credible intervals. Prior to decomposition, the original data were standardized to ensure that the empirical mean of each component was close to zero

Using the Gaussian likelihood of the model and the prior specification, maximum a posteriori (MAP) estimates of the model parameters were obtained with R 4.1.2 and rstan 2.21.5 (Carpenter et al. 2017). The posterior distribution of each component process of the additive decomposition (1) is known in closed form (Rasmussen and Williams 2005; Bilancia et al. 2021). Consequently, each posterior is fully determined by inserting MAP estimates of the model parameters into its expression. The estimated components are shown in Fig. 3, with the approximate posterior credible intervals.

**Fig. 4:**
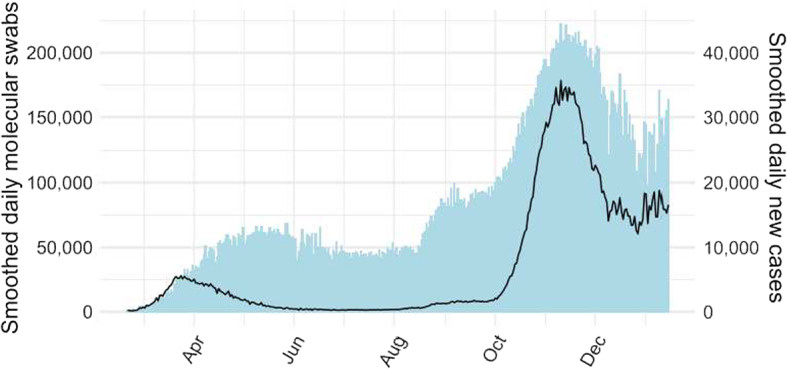
Smoothed series of daily new cases and molecular swabs. These series were estimated by subtracting from the standardized data the weekly periodic component with time-dependent amplitudes, which was estimated using an appropriately specified additive Gaussian process. The smoothed data were then transformed back to the original scale

The smoothed data are shown in Fig. 4. These smoothed versions were obtained by subtracting the estimated periodic component from the standardized data and then transforming back to the original scale.

## Methods

### Causal dynamic systems

To estimate causal impacts, we used the counterfactual approach proposed by Brodersen and co-authors (Brodersen et al. 2015), which is an extension of the difference-in-differences (DiD) design for observational data to time series data. The standard DiD design is based on a linear model of outcomes in the treatment and control groups and calculates the causal effect of a treatment or intervention by estimating the average change over time in the outcome variable for the treatment group compared to the average change over time for the control group (Bertrand et al. 2004; Abadie 2005; Angrist and Pischke 2008; Schwerdt and Woessmann 2020). The limitations of DiD designs with time series data are addressed using Bayesian state-space models in conjunction with a regression component to predict the time evolution of the counterfactual response. The sources of information needed to adequately estimate such an effect are (Abadie et al. 2010):

- The time series of the outcome before the intervention.
- The trajectories of other time series that predict the outcome before the intervention and whose value for predicting the counterfactual response is in the post-intervention phase. A key hypothesis is that the control time series themselves were not affected by the intervention. Assuming that the relationship between the control time series was stable in the pre-intervention phase, the algorithm learns the relationship between the treatment and control groups before the intervention and predicts the counterfactual series after the treatment.

### State-space models for causal effect estimation

The state-space time series models we used for causal analysis are (Durbin and Koopman 2012):

**MODEL A**. The local linear trend model, where the outcome variable ($y_t \in \mathbb R$) is described as the sum of a local time-dependent unobserved state ($\mu _t$) plus a Gaussian noise ($\varepsilon _t$):

9 $$\begin{aligned} y_t&= \mu _t + \varepsilon _t, \quad&\varepsilon _t \sim \mathcal N(0, \sigma ^2_\varepsilon ), \end{aligned}$$

10 $$\begin{aligned} \mu _{t+1}&= \mu _t + \nu _t + \xi _t, \quad&\xi _t \sim \mathcal N(0, \sigma ^2_\xi ), \end{aligned}$$

11 $$\begin{aligned} \nu _{t+1}&= \nu _t + \zeta _t, \quad&\zeta _t \sim \mathcal N(0, \sigma ^2_\zeta ). \end{aligned}$$

The latent state $\mu _t$ evolves as a random walk that depends on the sum of the state at the previous time and an additional latent variable ($\nu _t$), that is itself described by a random walk. As a result, the latent state variable is described by a local linear trend, which means that the intercept and slope of this trend change as the system evolves, adapting to upward and downward phases. This model was estimated on either the original or the smoothed data to evaluate the effects of the human-induced periodic component. $\square$

**MODEL B**. The local linear trend model with seasonality, where the outcome variable is described as the sum of a latent state variable $\mu _t$, which evolves exactly as in the previous case, and an additional latent state variable ($\gamma _t$) describing a non-stationary cyclical component, which models the periodic component with a period of $s=7$ days:

12 $$\begin{aligned} y_t&= \mu _t + \gamma _t + \varepsilon _t, \quad&\varepsilon _t \sim \mathcal N(0, \sigma ^2_\varepsilon ), \end{aligned}$$

13 $$\begin{aligned} \mu _{t+1}&= \mu _t + \nu _t + \xi _t, \quad&\xi _t \sim \mathcal N(0, \sigma ^2_\xi ), \end{aligned}$$

14 $$\begin{aligned} \nu _{t+1}&= \nu _t + \zeta _t, \quad&\zeta _t \sim \mathcal N(0, \sigma ^2_\zeta ), \end{aligned}$$

15 $$\begin{aligned} \gamma _{t+1}&= -\sum _{j=1}^{s-1}\gamma _{t+1-j}+\omega _t,\quad&\omega _t \sim \mathcal N(0, \sigma ^2_\omega ). \end{aligned}$$

This model was estimated based only on the original data to perform some kind of sensitivity analysis with respect to the model specification. $\square$  

Either MODEL A or MODEL B may contain contemporaneous control time series $x_t$ (or, briefly, covariates) that are accounted for via static coefficients. Such control time series are unaffected by the intervention and are essential to obtain accurate counterfactual predictions. As a matter of example, if:

$$\begin{aligned} x_t^\top = (x_{t1}, x_{t2},\ldots , x_{tk})^\top \in \mathbb R^k, \end{aligned}$$

denotes a general *k*-dimensional control time series, the local-level model (a special case of MODEL A with $\nu _t \equiv 0$) with control covariates is written as follows:

16 $$\begin{aligned} y_t&= \mu _t + \beta ^\top x_{t} + \varepsilon _t, \quad&\varepsilon _t \sim \mathcal N(0, \sigma ^2_\varepsilon ), \quad&\beta \in \mathbb R^k \end{aligned}$$

17 $$\begin{aligned} \mu _{t+1}&= \mu _t + \xi _t, \quad&\xi _t \sim \mathcal N(0, \sigma ^2_\xi ). \quad&\end{aligned}$$

### Bayesian posterior estimation of counterfactual effects

With a few algebraic manipulations, it is easy to see that all of the models defined above (either with or without control covariates) are a special case of the following Gaussian state-space model (GSSM), which for a general *d*-dimensional time series ($t=1,2,\ldots ,m$) has the following form (Durbin and Koopman 2012):

$$\begin{aligned} y_t&= Z_t \alpha _t + \varepsilon _t, \quad \text {observation-equation} \\ \alpha _{t+1}&= T_t \alpha _t + R_t \eta _t, \qquad \text {state-equation} \end{aligned}$$

where $y_t\in \mathbb R^d$ is observable, $Z_t \in R^{d \times m}$ and $\varepsilon _t \sim \mathcal N_d(0,H_t)$ with $H_t\in \mathbb R^{d \times d}$. The vector $\alpha _t\in \mathbb R^m$, which enters the equation of state, is a latent state vector which evolves in a Markovian way according to the matrices $T_t\in \mathbb R^{m \times m}$ and $R_t\in \mathbb R^{m \times r}$ with $r\le m$ and $\eta _t \sim \mathcal N_r(0,Q_t)$, where $Q_t$ is non-singular. The error terms are assumed to be serially and mutually independent. In general, the matrices $Z_t$, $H_t$, $T_t$, $R_t$, and $Q_t$ depend on a vector of unknown parameters $\varphi$. For example, the local-level model defined by the equations (16) and (17) with a single control time series ($k=1$) can be written in state-space form as follows:

18 $$\begin{aligned} \alpha _t = \begin{pmatrix} \mu _t \\ 1 \end{pmatrix},\quad Z_t = \begin{pmatrix} 1&\beta _1 x_{t1}\end{pmatrix}, \end{aligned}$$

with:

19 $$\begin{aligned} T_t \equiv T = \begin{pmatrix} 1 &{} 0 \\ 0 &{} 1\end{pmatrix},\quad R_t \equiv R = \begin{pmatrix} 1 \\ 0\end{pmatrix}, \quad H_t \equiv H = \sigma ^2_\varepsilon , \quad Q_t \equiv Q = \sigma ^2_\xi . \end{aligned}$$

Let $\alpha _{1:m}=(\alpha _1,\alpha _2,\ldots ,\alpha _n,\alpha _{n+1},\alpha _{n+2},\ldots ,\alpha _m)^\top$ denote the complete sequence of latent states. Assuming that the intervention occurs at time $t=n<m$, the $\alpha _{1:m}$ sequence includes both the pre-intervention subset, $\alpha _{1:n}=(\alpha _1,\alpha _2,\ldots ,\alpha _n)^\top$, and the post-intervention subset $\alpha _{(n+1):m}=(\alpha _{n+1},\alpha _{n+2},\ldots ,\alpha _m)^\top$. Accordingly, the pre-intervention observed values are denoted as $y_{1:n}=(y_1,y_2,\ldots ,y_n )^\top$, while the post-intervention counterfactual responses are denoted as $\tilde{y}_{(n+1):m}=(\tilde{y}_{n+1},\tilde{y}_{n+2},\ldots ,\tilde{y}_{m})^\top$. We distinguish these latent responses from the observations $y_{(n+1):m}=(y_{n+1},y_{n+2},\ldots ,y_h)^\top$, since the former represent what would have been observed in the absence of the intervention.

For the Bayesian posterior estimation of the GSSM parameters, we closely followed the specification proposed in Brodersen et al. (2015). The diffuse prior used for each member of the collection of inverse variances (collectively denoted as $\sigma ^{-2}_\bullet$) from MODEL B is:

20 $$\begin{aligned} \frac{1}{\sigma ^2_\bullet } {\mathop {\sim }\limits ^{\text {ind.}}} \text {Gamma}(a,b), \end{aligned}$$

with $a=10^{-2}$ and $b=10^{-2} s_y^2$, where $s_y^2$ is the unbiased sample variance of the target series. Combined with the Gaussian likelihood, the prior structure is the basis for a special Gibbs sampling algorithm used to simulate either from the joint posterior distribution $p(\varphi ,\alpha _{1:n} \vert y_{1:n})$ of the model parameters and latent states (in this notation, all model parameters are combined in the $\varphi$ vector), using only the observations in the pre-intervention period to train the model. Similarly, we use the joint posterior predictive distribution $p(\tilde{y}_{(n+1):m}\vert y_{1:n},x_{1:m})$ to estimate the counterfactual outcomes that would have been observed in the absence of the intervention. This predictive density depends coherently on the observations $y_{1:n}$ in the period before the intervention and on the full sequence of the control time series $x_{1:m}$ (Brodersen et al. 2015; Carter and Kohn 1994; Frühwirth-Schnatter 1994; Durbin and Koopman 2002). We used the implementation of this Bayesian posterior inference machinery provided by the library CausalImpact v.1.2.7, which implements the proposed algorithm in C++ and has a simple and intuitive R interface (Brodersen et al. 2022).

Samples from the posterior predictive counterfactual distribution can be used to obtain a posterior estimate of the pointwise causal effect:

21 $$\begin{aligned} \phi _{\textsf {point}}(t) = y_t - \tilde{y}_t,\quad t = n+1, n+2,\ldots , m. \end{aligned}$$

Other meaningful measures of causal effect, discussed in more detail in the next section, can indeed be defined. It is worth noting that observations from the post-intervention period are not used twice, both for training the dynamic models and for estimating the causal effects as defined by equation (21).

### Control time series

For the nationwide data, the time series of the number of molecular tests processed per day proved to be a suitable predictor of the outcome variable, the daily number of new cases. In a static linear regression model, we had $p < 0.001$ for the regression coefficient with the original and smoothed data. In addition, we found $R^2_{\text {LOOCV}} = 0.77$ and $R^2_{\text {LOOCV}} = 0.81$ in the two cases (LOOCV = Leave-one-out cross-validation, where $R^2_{\text {LOOCV}}$ is defined as the squared correlation coefficient between the observed dependent variable and the corresponding predicted values, one observation at a time removed from the training set and used for prediction; James et al. 2021). Theoretically, it is also possible to consider a relationship between the outcome series and the control time series based on a dynamic (rather than static) coefficient, as explained in Brodersen et al. (2015). We considered the use of this option essentially unnecessary given the substantial stability in the pre-intervention periods used. For this reason, the daily number of swabs enters the causal state-space model via a static coefficient, as in the equation (16).

The question of whether the number of daily swabs can be included in the causal model as a control time series requires further discussion. If we were to argue in the traditional terms of time series econometrics, we might indeed assume that the two daily time series (swabs and positive counts) influence each other or have a feedback effect, but this influence is indeed limited. Consider a phase when the number of cases increases due to the increase in viral circulation. When this increase is very large (as in the second wave), the authorities respond by strengthening the surveillance system and increasing the number of swabs. Of course, the increase in the number of swabs leads to the detection of more new positive cases, but this effect is very limited, since most of new cases are detected due to the exponential increase in viral circulation (with the consequent rapid increase in the positivity rate). That this is indeed the case is confirmed by the fact that by the end of November, the number of daily swabs had increased by a factor of 4 (compared to the post-lockdown phase), while the number of cases had increased by a factor of 300 (from about 100 to almost 40,000). Thus, we can be reasonably confident that the number of daily swabs is not affected by the intervention, even if only approximately.

In the analyses conducted with regionally disaggregated data, the average daily temperature of the regional capital was included as an additional covariate for each Region. In recent months, studies documenting the inverse relationship between the occurrence of COVID-19 and the timing of key meteorological variables have been published frequently. These papers have focused on establishing robust inverse correlations between meteorological factors and the transmission of COVID-19 (Mecenas et al. 2020; Rouen et al. 2020; Hu et al. 2021). While it is clear that the interventions do not directly lead to temperature changes, the fluctuations observed in this variable from one season to the next are so large that the local temperature time series cannot be considered as at least second-order stationary. These fluctuations are particularly evident in the case of the second intervention, the reopening of the schools, since it occurred during a period of transition from the summer to the fall season. In the post-intervention phase, a rapid decrease in temperature was observed. This means that this control time series is not useful for estimating the causal effect (21), but rather can be considered as an effect modifier. When temperature decreases, the estimate of the counterfactual response $\tilde{y}_t$ tends to increase, and thus, the estimate of the causal effect $\phi _{\textsf {point}}(t)$ tends to decrease, because some of the observed variation is due to the temperature change rather than to the intervention itself. Of course, when the temperature level is high, the reverse reasoning applies.

## Results

### Nationwide level

For the two interventions whose causal effect we want to estimate, we considered the following two subdivisions:

- DPCM June 11, 2020 (effective from June 15, 2020): Pre-intervention period from February 21, 2020 to June 15, 2020; Post-intervention period: from June 16, 2020 to September 14, 2020.
- Reopening of schools: Schools reopened at slightly different times depending on the Region. However, for the nationwide analysis, we used the date that most Regions opened the new school year: September 14, 2020. The pre-intervention period ranged from February 21, 2020 to September 14, 2020. The post-intervention period ranged from September 15, 2020 to January 14, 2021.

In addition to the pointwise causal effect (21), we also calculated the cumulative causal effect:

22 $$\begin{aligned} \phi _{\textsf {cumul}}(t) =\sum _{s=n+1}^t \phi _{\textsf {point}}(s), \quad t=n+1,n+2,\ldots ,m. \end{aligned}$$

**Fig. 5:**
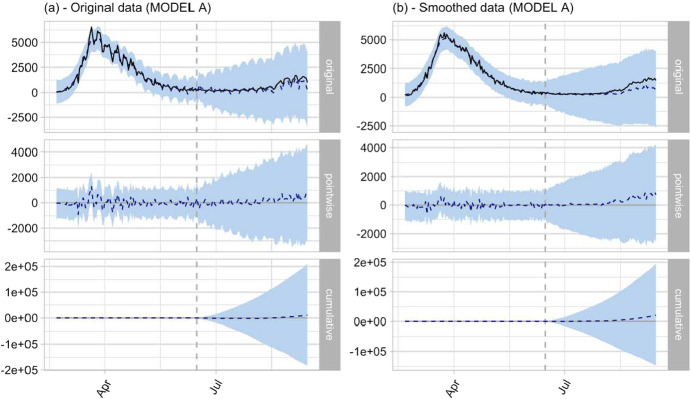
Trajectories of causal effects for the June 11, 2020 intervention (the DPCM that allowed reopenings). The pre-intervention period is February 21, 2020, to June 15, 2020, and the post-intervention period is June 16, 2020, to September 14, 2020. Analyses were conducted using either the original data (**a**) or the smoothed data (**b**), and MODEL A. Approximate 95% posterior credible intervals, shaded in light blue, were calculated for all time points *t* in both the pre- and post-intervention periods

For both (21) and (22), central posterior credible intervals (PCIs) were calculated at the 95% level. Given the observed data, the posterior estimate of a causal effect is a random variable, and has a probability of 95% within its PCI, given the evidence of the observed data. For an example of the trajectories of the point and cumulative causal effects calculated using the nationwide data, see Fig. 5.

PCIs can also be used to calculate a third measure of causal effect that can provide important clues about the biological plausibility of estimated causal effects. This new measure is defined as:

23 $$\begin{aligned} \textsf {lag} = \min _t \Big \{t\in n+1,n+2,\ldots ,m:\, \left( \inf \text {PCI}\left[ \phi _{\textsf {cumul}}(t) \right] \right) > 0 \Big \}, \end{aligned}$$

i.e., the lag is equal to the number of days between the intervention and the first day that the PCI of the cumulative causal effect (22) does not contain 0. In this regard, it is important to note that both state-space models used have Gaussian likelihood, whereas our data are counts. The use of such an approximation means that the predictions of the counterfactual model may take negative values at certain stages when the incidence is low. However, this problem only arises when the estimates of the causal effect turn out to be not statistically significant and can therefore be safely ignored.

Trajectories of causal effects are not particularly useful for the purposes of quantitative synthesis. Therefore, to summarize and present the results obtained, we considered the posterior summaries presented in Table 1, which include the average and total causal effect for the entire post-intervention period. During the post-intervention, the outcome variable had an average daily value of approximately 567 new cases. Without the intervention, we would have expected an average outcome of 460 cases, with a 95% PCI of this counterfactual effect given by ($-1672$ to 2606). The absolute effect is obtained by subtracting the counterfactual prediction from the actual average. This effect equals 107, with a 95% PCI given by ($-2039$ to 2238). In other words, although numerically an average of 107 additional cases per day can be attributed to the intervention, the 95% PCI is a wide interval that includes zero, and therefore, the observed effect cannot be considered significant from a Bayesian posterior perspective (see Kelter 2020, for a good overview of Bayesian posterior significance as opposed to classical frequentist significance). Comparable nonsignificant results were obtained when the Bayesian state-space model was trained on the smoothed data. Moreover, the probability that these effects are purely random is $p=0.46$ for both the original and smoothed data. This particular Bayesian *p* value is based on the posterior predictive distribution of the counterfactual outcome that would be expected in the absence of the intervention. The actual values are then compared to this posterior distribution, and *p* represents the tail-area probability under the calculated posterior that a positive effect is at least as extreme (away from the expected value) as the observed (Meng 1994; de la Horra and Rodriguez-Bernal 2001). At $p=0.46$, the probability of randomly obtaining a positive effect at least as large as the observed one is quite high. This confirms that causal effects for the first intervention can be considered spurious and not significant in the sense defined above. Similarly, the total effect is 9693 new cases in the post-intervention period, with 95% PCI = ($-188{,}588$ to 203,700) and $p=0.38$ with the original data and 25,496 with 95% PCI = ($-147{,}462$ to 196,291) and $p=0.38$ with the smoothed data.

**Table 1:** Posterior summaries of post-intervention effects

	Actual	Pred	Lower	Upper	SD	Abs. effect	Lower	Upper	SD	*p*	Lag (*d*)
Model A (avg.)	567	460	$-1672$	2606	1084	107	$-2039$	2238	1084	0.46	
1st interv.—original data											
Model A (total)	51,562	41,869	$-152{,}138$	237,150	98,608	9693	$-185{,}588$	203,700	98,608	0.46	NA
1st interv.—original data											
Model A (avg.)	656	375	$-1501$	2276	949	280	$-1620$	2157	949	0.38	
1st interv. —smoothed data											
Model A (total)	59,657	34,161	$-136{,}634$	207,119	86,400	25,496	$-147{,}462$	196,291	86,400	0.38	NA
1st interv. —smoothed data											
Model B (avg.)	567	398	$-1688$	2556	1083	168	$-1990$	2254	1083	0.44	
1st interv. —original data											
Model B (total)	51,562	36,230	$-153{,}578$	232,631	98,513	15,332	$-181{,}069$	205,140	98,513	0.44	NA
1st interv. —original data											
Model A (avg.)	16,808	2243	106	4449	1125	14,566	12,359	16,702	1125	$<0.001$	
2nd interv. —original data											
Model A (total)	2,050,585	273,588	12,920	542,754	137,266	1,776,997	1,507,831	2,037,665	137,266	$<0.001$	21
2nd interv. —original data											
Model A (avg.)	16,698	2066	154	3989	986	14,631	12,709	16,544	986	$<0.001$	
2nd interv. —smoothed data											
Model A (total)	2,037,146	252,105	18,751	486,662	120,334	1,785,041	1,550,484	2,018,394	120,334	$<0.001$	19
2nd interv. —smoothed data											
Model B (avg.)	16,808	1905	$-344$	4191	1166	14,903	12,617	17,152	1166	$<0.001$	
2nd interv. —original data											
Model B (total)	2,050,585	232,426	$-41{,}950$	511,352	142,268	1,818,159	1,539,233	2,092,535	142,268	$<0.001$	20
2nd interv. —original data											

For the 1st intervention, the pre-intervention period is from February 21, 2020, to June 15, 2020, and the post-intervention period is from June 16, 2020, to September 14, 2020. For the 2nd intervention, the pre-intervention period is from February 21, 2020, to September 14, 2020, and the post-intervention period is from September 15, 2020, to January 14, 2021. *Actual*: actual average daily number or actual total number of new cases during the post-intervention period. *Pred*: the corresponding counterfactual outcome predicted by the Bayesian state-space model. *Lower* and *upper*: lower and upper bounds, respectively, of central 95% posterior credible interval (PCI) of the counterfactual outcome. *sd*: posterior estimate of the standard deviation of the counterfactual outcome. *Abs. effect*: difference between the actual outcome and the counterfactual outcome (with associated 95% PCI lower and upper bounds, and standard deviation). *p*: Bayesian tail-area probabilities calculated under the posterior predictive distribution of the counterfactual outcome in the absence of the intervention. It represents the probability that a positive effect will be at least as extreme (away from the expected value) as the observed one. For example, $p < 0.001$ means that if the intervention had no effect on the outcome variable, the probability of obtaining by chance a positive effect at least as large as the observed one is less than 1/1000. *Lag*: measured in days, it is equal to the number of days between the intervention and the first day that the PCI of the cumulative causal effect does not contain zero. It was not computable for the first intervention, since the corresponding PCIs always contained zero for each time point in the post-intervention period

On the other hand, the situation is quite different when the effects of reopening schools are analyzed. From Table 1, we can conclude that the total effect after the intervention is 1,776,997 new cases based on the original data, with a 95% PCI = (1,507,831 to 2,037,665) and $p < 0.001$. In other words, if the intervention had no effect on the response variable, the probability of this effect occurring by chance would be less than 1/1000. These impressive figures are confirmed in all the scenarios presented in Table 1. It should also be noted that the number of new cases attributable to the second intervention should not be confused with the number of infected students in the post-intervention period. Rather, it is the number of new cases observed in the general population that are attributable to the chain of events set in motion by the reopening of schools and that would not have occurred in the absence of this intervention.

The causal statistic (23) is obviously not computable for the first intervention, since the corresponding PCIs did not exclude 0 for each time point $t=n+1,n+2,\ldots ,m$. For the second intervention, it is important to note that the estimated average lag was 20 days (an average value for the three data analysis scenarios). This estimate was very close to the incubation period of COVID-19 disease, which is thought to be 14 days, with a median time of 6–7 days from exposure to onset of symptoms (Guan et al. 2020) with one study reporting that 97.5% of individuals with COVID-19 had symptoms within 11.5 days of SARS-CoV-2 infection (Lauer et al. 2020). In other words, the estimated lag is consistent with the hypothesis that the intervention restarted the viral circulation and that, after a period approximately twice as long as the maximum incubation period, a sufficiently large number of individuals became symptomatic and tested positive for the molecular PCR-RT test.

### Regional level

The nationwide data analysis is inconclusive. The national data are a sum of regional data, so the lag estimated at the national level may mask very different patterns across Regions. Moreover, analyses at a narrower spatial level may be enriched with confounding variables (e.g., meteorological variables) that, by their nature, cannot be included in the causal model estimated at the national level.

**Fig. 6:**
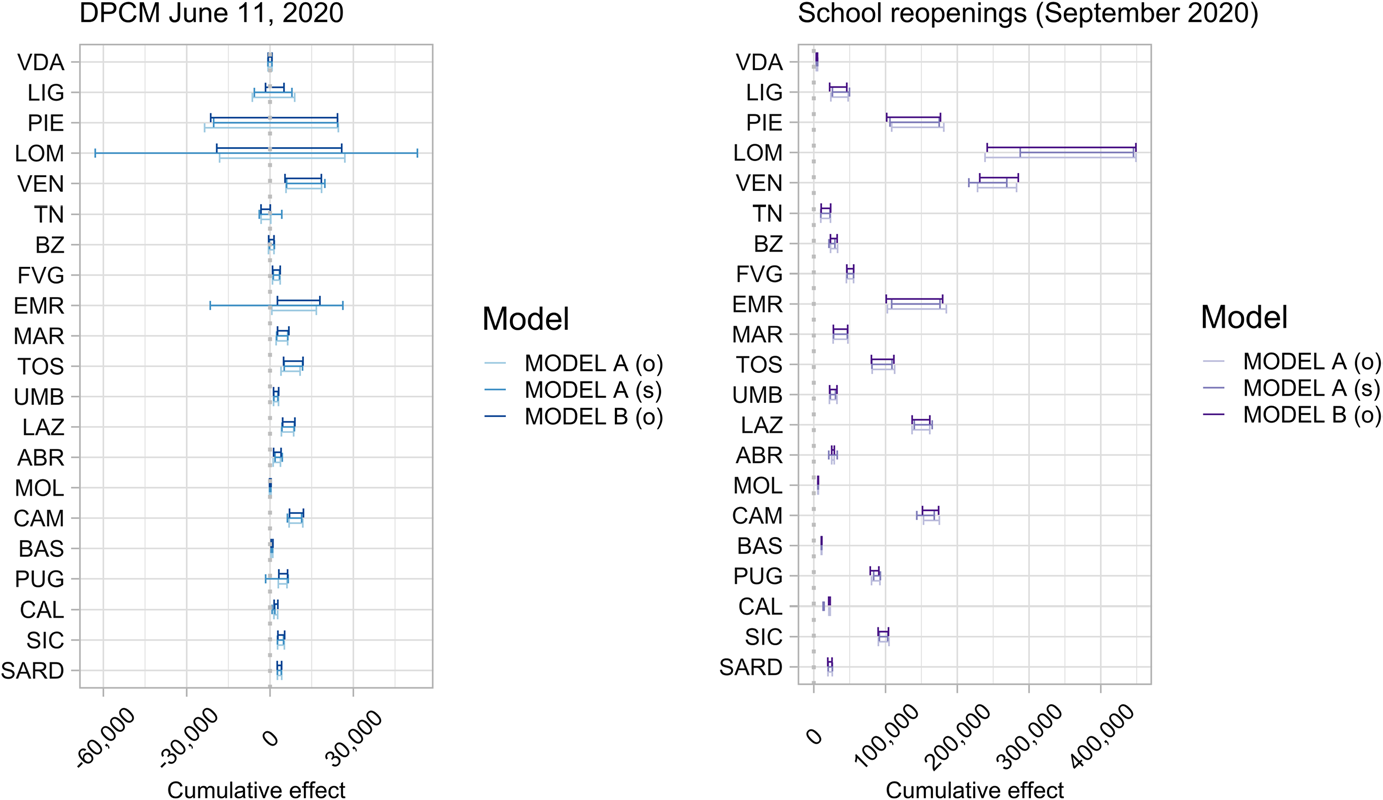
95% PCI of estimated total causal effects by Region, for each of the three data analysis strategies (o = original data, s = smoothed data). Legend: VDA = Valle d’Aosta; LIG = Liguria; PIE = Piemonte; LOM = Lombardia; VEN = Veneto; TN = Provincia autonoma di Trento; BZ = Provincia autonoma di Bolzano; FVG = Friuli Venezia Giulia; EMR = Emilia Romagna; MAR = Marche; TOS = Toscana; UMB = Umbria; LAZ = Lazio; ABR = Abruzzo; MOL = Molise; CAM = Campania; BAS = Basilicata; PUG = Puglia; CAL = Calabria; SIC = Sicilia; SARD = Sardegna

Regarding the June 11, 2020, DPCM, Fig. 6 shows that all Regions of the northwestern macro-area had nonsignificant causal effects (Valle d’Aosta, Liguria, Piemonte, Lombardia). These Regions reported the highest number of confirmed cases relative to the resident population during the first outbreak, so the fluctuations observed from June 15, 2020 onward should not be considered significant compared with the overall pattern of the preceding months. Uncertainty in the predictive distribution includes variability over the entire pre-intervention period, so fluctuations in the post-intervention period that are not at least quantitatively comparable are not considered significant (in the Bayesian sense defined above). Even in the northeastern Regions where the causal effect was significant, such as Friuli Venezia Giulia, the lag was longer on average than in the southern Regions, because a longer period, at least five times the average incubation period, was required before the cumulative causal effect became detectable. In contrast, in the southern Regions, which had relatively low infection rates during the first wave, the increase in incidence rates observed after the relaxation of restrictive measures quickly became significant. As for the reopening of schools, the causal effect proved strong in all Regions, with average levels roughly proportional to the resident population.

We also performed a meta-analysis of the regression coefficients for each of the two input variables (the daily number of molecular tests and the daily temperature of the regional capital), considering all regression coefficients estimated for each Region and the three data analysis strategies (for a total of 63 estimates). For the first intervention, using a fixed-effects model with mean difference as the effect size (Borenstein et al. 2010), we obtained a pooled effect for temperature of $-0.5010$ (95% CI: $-\,0.5223$ to $-0.4798$; $p<0.001$), whereas for the number of molecular tests we obtained 0.1749 (95% CI: 0.1543 to 0.1955; $p < 0.001$). Similarly, for the second intervention, we obtained $-0.1150$ (95% CI: $-0.1353$ to $-0.0948$; $p < 0.001$) for the daily temperatures and 0.1152 (95% CI: 0.1034 to 0.1269; $p < 0.001$) for the daily number of molecular tests. As expected, these results seem to confirm the existence of an inverse relationship between the daily mean temperature and the daily number of new cases.

**Fig. 7:**
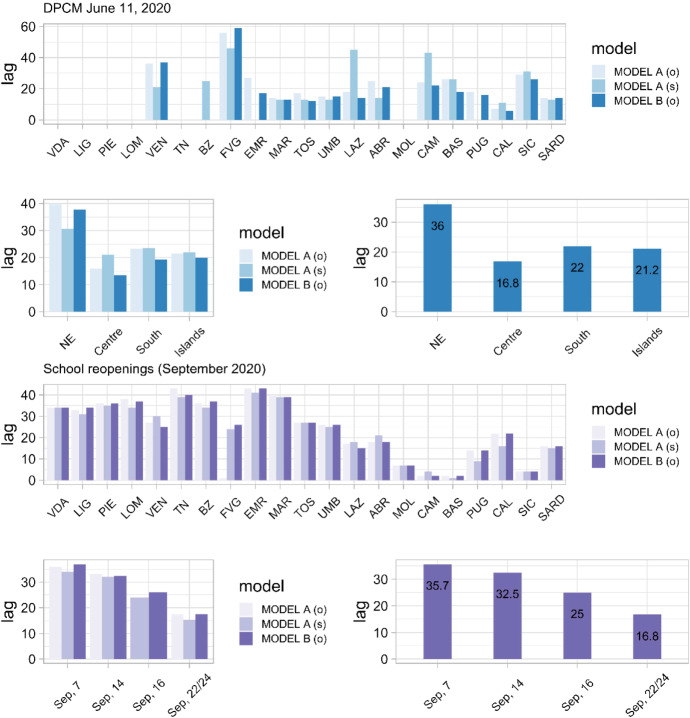
(Top panel—First intervention) Estimated lag by Region and data analysis strategy (o = original data, s = smoothed data), averaged for each geographic macro-area. Legend: NE = Veneto + Provincia Autonoma di Trento + Provincia Autonoma di Bolzano + Friuli Venezia Giulia; Centre = Emilia Romagna + Marche + Toscana + Umbria + Lazio; South = Abruzzo + Molise + Campania + Basilicata + Puglia + Calabria; Islands = Sicilia + Sardegna. (Bottom panel—Second Intervention) Estimated lag by Region and data analysis strategy (o = original data, s = smoothed data), averaged for each school reopening date

The spatial distribution of the lag and its temporal pattern were also examined in more detail. Specifically, for the first intervention, we averaged the regional estimates to obtain a single average value for each geographic macro-area (results are shown in Fig. 7, top panel). As noted earlier, for most northern Regions the lag was not estimated because the cumulative causal effect was not significant. Only in three Regions of the northeastern macro-area it was possible to calculate the lag, with an average value of 36 days. This value can be explained by the relative comparison with the first outbreak, as explained above. In the other central and southern Regions, the estimated lag was shorter. The highest average lag of 22 days occurred in the southern macro-region. Overall, these results seem to suggest that the causal relationship between relaxation of restrictive measures and resumption of virus circulation is markedly robust, especially in the southern Regions.

Far more uncertain, however, are the conclusions that can be drawn regarding the reopening of schools. To avoid biasing the conclusions too much in favor of the hypothesis of a causal relationship between reopening of schools and recurrence of infection, we excluded from the calculation of group averages all cases for which the estimated lag was equal to or less than 7 days. In addition, Regions that reopened on September 22 or 24 were combined into a single group because the individual subgroups were too small to draw statistically stable conclusions. Even when these issues were explicitly accounted for, Fig. 7 (bottom panel) shows that there is a clear inverse relationship between the lag and the reopening dates. For Regions that opened earlier, the lag was longer, while it became shorter toward the end of September 2020. Thus, we can conclude that the dynamics of contagion during the second outbreak show a sudden acceleration that is not synchronized with the reopening of schools, but is concentrated in the window between October 10 and 17, 2020, for the entire country. This unexpected result raises complex interpretive questions regarding the presumed causal effect of school reopening.

### The effect of omitting interventions

**Fig. 8:**
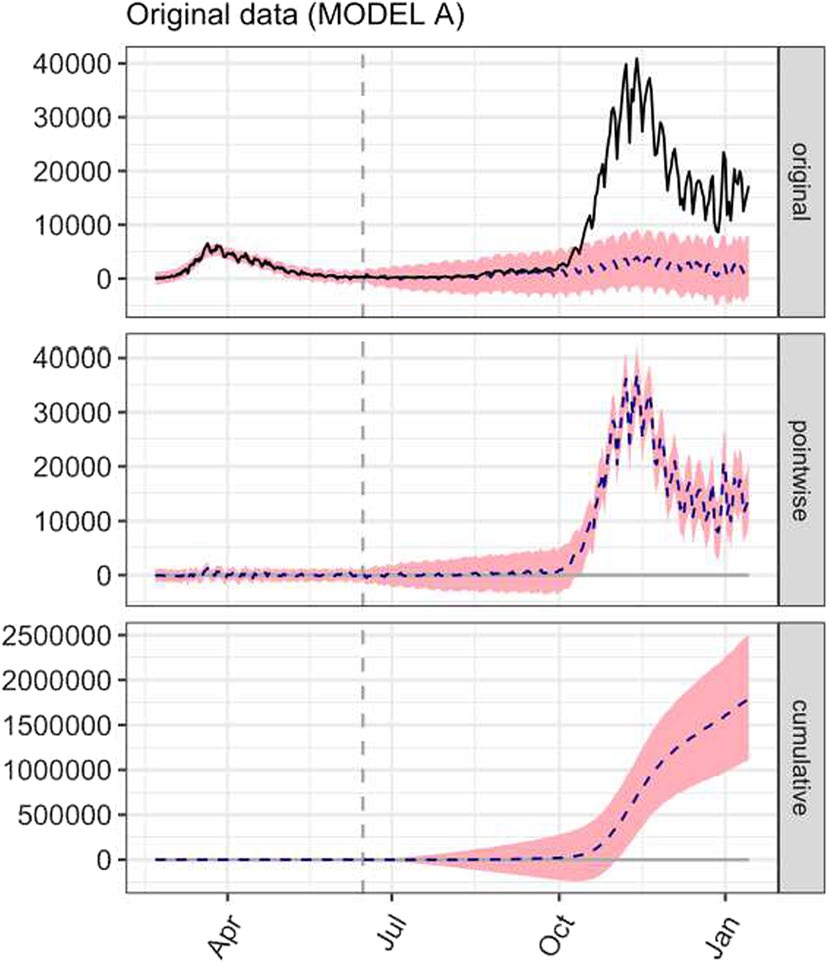
Alternative causal impact analysis for the first intervention (the June 11, 2020 DPCM that allowed reopenings) using MODEL A and nationwide data. The pre-intervention period is February 21, 2020, through June 15, 2020, and the post-intervention period is June 16, 2020, through January 14, 2020. The second intervention was omitted, and the post-intervention period was extended to include the entire second outbreak

Figure 8 shows a nationwide analysis for the June 11, 2020, DPCM, with the post-intervention period extended to January 14, 2021, including the entire second outbreak and omitting the second intervention. As it can be seen, also in this case the causal effect is significantly different from the corresponding counterfactual. However, the estimated lag was about 140 days. The cumulative causal effect became significant in early November, but the high and biologically implausible value of the estimated lag suggests that we neglected later events that are most likely causally related to the observed increase in the number of cases. Thus, an excessively large and biologically implausible lag weakens the hypothesis of a true causal relationship between the intervention and the subsequent increase in observed cases. When we analyze the causal impact curve from November 2020, we can also observe a significant decrease in its slope, which is due to the reintroduction of new restrictive measures by another DPCM of November 3, 2020. Thus, if we exclude an intervention that occurred in the post-intervention period, any significant effect is still included in the causal impact curve. In choosing the pre- and post-intervention time periods, we considered this basic point to avoid confusion and identification problems between effects attributable to the June 11, 2020, DPCM and those attributable to the reopening of schools in mid-September 2020.

## Discussion

In this work, we have attempted to estimate some selected causal effects on the dynamics of COVID-19 infection using a structural Bayesian model proposed in Brodersen et al. (2015). This method generalizes the concept of DiD design and uses a structural time series model to infer the causal effects of a point-in-time intervention. The model learns the relationship between the treatment and control groups before an intervention and predicts the counterfactual series after the treatment. Within the proposed Bayesian hierarchical specification of the model, the posterior distribution of the counterfactual time series can be simulated and estimated using an appropriate MCMC algorithm. The causal effect is then estimated by subtracting the predicted from the observed post-intervention time series. Although the study of causality in a dynamic setting can in principle be performed using other models for time series analysis (e.g., to name a few: vector autoregressive models, hidden Markov processes, or deep neural networks; see Moraffah et al. 2021, for a comprehensive overview), the approach proposed by Brodersen et al. (2015) is based on state-space models. In its structural form, this class of models allows the selection of appropriate components for trend, seasonality, and static regression for the set of contemporaneous control time series. The latent state vector $\alpha _t$ and associated model matrices can be assembled from standard templates of component submodels to capture features of the data. There are several well-described state-space models in the literature that can be used as needed to capture non-stationary or periodic components (as in our case) without special modeling effort, allowing the data analyst to focus solely on analyzing and interpreting the results. The model we have chosen is conditioned by the particular shape of our data. However, it is clear that other specifications might be appropriate for other applications.

Regarding the specific problem of explaining the results obtained, we first address the impact of the DPCM of June 11, 2020. The estimated lags are consistent with the presence of a causal effect for the increase in contagion in the central and southern Regions of the country, where the impact of easing of restrictions became significant after a time interval that was about three times longer than the median incubation period. The effects in the northern Regions of the country were more nuanced with respect to the amplitude of the first outbreak, resulting in a nonsignificant causal effect or a significant effect with an estimated lag that was significantly higher than that estimated for the southern Regions. To better analyze the exit strategy from the first lockdown, we used a five-factor model recently presented in the literature (Han et al. 2020).

The first factor is the availability of indicators to monitor the epidemiological situation. Although in June 2020 the sophisticated indicator system was not yet available, which from December 2020 allowed the classification of Regions into different colors according to the risk of infection, the Italian surveillance capacities (greatly expanded and improved during the second outbreak) undoubtedly allowed a timely response to the increasing contagion, even if they were not sufficient to maintain the low level of virus circulation observed in July 2020.

Another factor is the capacity of the public health system in terms of testing, detection, and isolation. In Italy, after initial difficulties, the situation improved in April 2020, also thanks to the initiative of 290 representatives of the national scientific community, all of whom wrote a letter to the Prime Minister to include new laboratories, including private ones, in the research network on COVID-19 and to increase the number of daily molecular tests. After the initial phase in which molecular testing was reserved for symptomatic individuals, the number of daily tests increased sharply from April 2020, in part due to the introduction of drive-through testing. Contact tracing substantially dampened the number of new infections, at least until late September 2020, when the absolute number of new daily infections was not too high. What does not parallel the first outbreak, however, is the rate at which the number of new cases increased at the beginning of the second outbreak. The curve of daily infections rose abruptly in October 2020, and contact tracing measures that had worked well from June 2020 to September 2020 were completely overwhelmed by this new outbreak.

The third factor, health system capacity, is of marginal importance for understanding the epidemiological phase from June 2020 to September 2020. Although this aspect was certainly not directly responsible for the new wave of infections, there was a significant decrease in the number of intensive care units and a decrease in the number of units dedicated to COVID-19 patients from June 2020. This error will manifest itself during the second outbreak.

The fourth factor is the control of national borders. For Italy, the situation after June 15, 2020, was characterized by the reopening of the Schengen area borders on June 30, 2020. The only measures that took effect in July 2020 were a complete ban on travel to and from 13 non-European countries considered high-risk areas. In Italy, a regulation requiring molecular testing for citizens returning from Greece, Spain, Malta, and Croatia, popular summer travel destinations, did not go into effect until August 12, 2020. However, it is clear that while the significant lack of protection at the external borders may have played a role in the rising epidemiological phase from mid-August, it did not play a role in the dynamics observed in the southern Regions, where, as noted above, there was a statistically significant acceleration of infection just three weeks after the DPCM came into effect.

Finally, the last and most important factor was community engagement, i.e.: (a) adherence to safety guidelines for physical distancing and wearing masks during the post-lockdown phase; (b) precautions in schools and workplaces; and (c) communication to ensure cooperation with the public. We will return to the second point shortly. Regarding the first point, the best practices that characterized the first lockdown were quickly and universally abandoned with the reopenings authorized by the DPCM. Although as of May 2020 it was already mandatory to wear masks indoors (e.g., on trains) or in outdoor places where the minimum distance of one meter could not be maintained, a paradigmatic survey conducted by Coldiretti in August 2020 found that more than one in four Italians (27%) reported not wearing a mask or not following all the safety measures prescribed by the pandemic (Coldiretti 2020). In addition, easing of restrictions after June 15 led to a “mass effect” as several Regions (e.g., Campania from June 12, Toscana from June 13, and Puglia and Sicilia from June 15) allowed summer activities such as discos to resume (in some Regions, such as Lombardia, these activities were closed during the summer) due to concessions granted by the DPCM. All these bad practices were exacerbated by the claim of some authoritative members of the scientific community that COVID-19 had become irrelevant in terms of its clinical hazard. Claims that now seem facile in light of subsequent events that have resulted in at least 70,000 additional deaths.

The reopening of schools and the link to SARS-CoV-2 transmission in the population were immediately the subject of heated debate both in Italy and elsewhere in the world. School closures have well-documented effects on social interaction and psychological well-being of students, especially adolescents, as well as problems with learning performance through distance education (de Figueiredo et al. 2021; Pokhrel and Chhetri 2021). In Italy, similar to K-12 schools, educational organization is divided into cycles that include kindergarten (ages 3 to 5), elementary school (ages 5 to 10), middle school (ages 11 to 13), and high school (ages 14 to 18), with compulsory education from ages 6 to 16. When schools reopened in September 2020, a sophisticated protocol was put in place to mitigate the risk of transmission within the school. It included strict control of body temperatures, hand hygiene at the entrance to school buildings, and mandatory use of face masks for all teachers and technical staff, as well as for students in common areas. Wearing a face mask while sitting at a desk was mandatory for all students beginning November 6, 2020, while this mandate applied only to high school students (and all teachers) during the critical period in October 2020. In addition, interpersonal distancing of 1 Meter was mandated for students sitting at desks. Many schools were able to use special desks to ensure better mobility and compliance with the interpersonal distancing. Finally, the protocol included the elimination of physical activities in schools, frequent ventilation of classrooms, and the reduction of instructional time. (one hour of instruction was reduced to 45 or 50 minutes for distance learning). Monitoring measures in schools were also significantly improved. For example, it was mandated that any new report of SARS-CoV-2 positivity in students, teachers, or school staff be handled by school principals and local National Health Service units in close collaboration to conduct rapid secondary screening for close contacts of the positive case and impose mandatory quarantine (with return to school after 10 days if the test was negative or after 14 days if no molecular swab was performed).

As claimed in a recent study (Gandini et al. 2021), this protocol kept transmission within Italian schools at a very low level, with a secondary transmission rate of less than 1%, regardless of whether the index case was a student or a teacher. These estimates were obtained from contact tracing data in a sample of approximately 6,000 schools between November 23 and December 5, 2020, and were confirmed in a smaller survey (reported in the same publication) of 339 schools in the province of Verona (Veneto Region). These results are consistent with other contact tracing studies available in the literature and suggest that transmission between students is a relatively rare event (Dub et al. 2020) and that students are not the primary source of exposure to SARS-CoV-2 among adults within schools (Heavey et al. 2020; Macartney et al. 2020). However, evidence of secondary transmission between students appears to be somewhat higher among high school students (Goldstein et al. 2021).

The Italian study cited earlier, using ISTAT data for the period between September 12 and November 8, 2020, also reported that the incidence of positive cases among high school students was relatively low at 98/10,000 (slightly below the national average), whereas among teachers and non-teaching staff it was 220/10,000 (about twice the national average). These results are also consistent with the literature, in which the majority of available studies report that incidence among students in schools is lower or at most equal to that in the general population and that few cases are reported in schools when the overall incidence is low, whereas an increase in community transmission is immediately reflected in an increase in incidence in schools (Ismail et al. 2021).

Although these data support the conclusion that transmission of the virus within school buildings is reduced by safety protocols, the role of schools in the transmission of the virus needs further investigation. To document the supposed irrelevance of reopening schools in maintaining virus circulation in the community, the same previously cited Italian study reported that in the Veneto Region, between August 28 and October 24, 2020, incidence in the 45- to 49-year-old age group increased from $\sim$2/10,000 to $\sim$35/10,000. The authors also noted that during the period between August 28, 2020, and September 6, 2020, incidence was high in the 45- to 49-year-old and 25- to 39-year-old age groups and remained at very low levels in all other age groups. However, the new positive cases diagnosed in this time window are better explained by exposures in the population that occurred in the second half of August 2020 (taking into account the distribution of the incubation period) and thus most likely related to tourist travels that occurred throughout the country at least until the end of August 2020. Using data from the same study, it can also be noted that from the beginning of October 2020, there was an increase of incidence in all age groups (Fig. 4, page 8), with the exception of children up to 9 years of age, in whom incidence remained essentially stable (probably due to the undercoverage typical of these age groups, which is due to lower expression of ACE-2 receptors—the entry port of the virus into tissues—resulting in lower susceptibility to severe forms of the disease). Thus, there is a significant association between incidence rates in the school-aged group (particularly 10- to 18-year-olds) and those in other age groups, which is consistent with findings in the literature and does not confirm but does not rule out a direct role for virus circulation in school buildings in enhancing virus circulation in the community. We address this important point in more detail in the next section, also in light of recent literature.

It remains to explain the inverse relationship between the lag and reopening dates shown in Fig. 7. We believe that the inverse effect of temperature on virus circulation should not be forgotten (Sebastiani and Palù 2020). The first reopenings occurred between September 7 and 14 in the northern Regions, a period characterized by a mild summer climate that still encouraged people to maintain interpersonal distance and allowed full application of safety protocols, including frequent ventilation of classrooms. High temperatures in September 2020 made it difficult for the virus to spread. Schools reopened on September 14, but incidence rates remained stable across the country. Weather conditions changed abruptly in the first days of October 2020. Eight storms moved through during that month, most of them in the first two weeks. Some of them proved to be quite intense, especially in early October, when the southernmost branch of a cloud system from the English Channel area reached our northwestern Regions, causing extreme precipitations (Abelli 2021). The decrease in temperatures during the first decade of October 2020 coincides with an increase in viral circulation in all age classes. It is also important to consider that in September 2020, the transportation system was not improved to prevent gatherings of students and commuters who often had to share tight spaces for more than 30 minutes. The use of strict protocols guarantees containment of viral circulation within schools, but offers no guarantee outside schools, where social habits and lower compliance may contribute to rapid increases in viral circulation (also considering the higher prevalence of asymptomatic individuals in the 18-year-old age group).

## Conclusions and future work

When the relaxation of restrictive measures was first granted in May 2020 and subsequently extended by the June 11 DPCM, the country did not abruptly return to “normal” life. Schools remained closed until the end of the school year, and many jobs (especially in the public sector) continued to be done remotely. We can say that the first real upswing in the movement of people occurred with the summer vacations of 2020. If we look at the absolute number of cases (it is not immediately clear from the graph, but we have to look at the data), we observe a new increase already at the end of July 2020, while the minimum was reached on June 23 with 122 confirmed cases. However, as noted above, the maintenance of some safety measures (such as wearing a mask indoors and keeping a safe distance), the fact that many jobs continued to be done remotely, and the complete closure of schools and universities implied that the rebound in the number of new positive cases (especially in northern Regions) was limited compared with the levels observed in the first wave.

However, we would also like to point out that from August 16, 2020, due to the above-mentioned increase in cases observed at the end of July, restrictive measures were ordered again: Discotheques were closed for the entire summer, and the wearing of masks was made mandatory in outdoor assembly areas from 6:00 pm until 6:00 am the next day. All of this makes it clear that despite the reopenings, there was not a full resumption of all economic/social activities and normal life in June, July, and August 2020. For the reasons explained so far (including the summer season and the ease of maintaining a critical interpersonal distance), the risk of contagion has been largely reduced despite the enactment of the DPCM. Thus, it is not entirely justified to attribute the lack of a causal effect to a sharply declining pandemic curve. It is a more complex and regionally differentiated situation that we have tried to describe. It is certainly the result of balanced decisions taken by the Italian Prime Minister, who has tried to put the economic and social life of our country back on track, but without exaggerating the lack of prudence.

Regarding the reopening of schools in September 2020, it is very likely that they acted as a rapid amplifier of virus circulation in the community once weather conditions were favorable for a more sustained general circulation. In the absence of a vaccination program, we believe that in any scenario involving respiratory illness with high replication rates, reasonable containment strategies should include, at a minimum, ongoing screening in schools through the mass administration of rapid antigenic/molecular tests to suppress the development of a potential cluster and ensure that contact tracing activities can continue effectively. However, in times of rapid increase in virus circulation, when tracing measures lose their effectiveness, we believe the precautionary principle justifies resorting to extraordinary measures such as suspension of classroom teaching activities.

The findings reported in this paper are also consistent with some important research on the first outbreak. Italy was the first European country to close schools in response to the pandemic emergency. The first cases were diagnosed on February 21, 2020, and schools and universities at all levels were closed on March 4, just 13 days after the first cases and 12 days before the total lockdown (in effect since March 16). The school closures were thus the first wide-reaching NPI applied in Italy, and subsequently similar measures were taken in all European countries and around the world. In this context, the authors of a large study that examined 41 countries for the period up to the end of May 2020 (Brauner et al. 2021) concluded that the most significant effects on the decline of $R_t$ were due to school and college closures (–38%), social distancing measures (–42%), and nonessential business closures (–18%). In contrast, the impact of stay-at-home orders was comparatively smaller (–13%).

Regarding more recent data after the second wave, an important paper was recently published by Manica et al. (2022), which concluded that a higher proportion of infected individuals causing onward transmission was found among students based on a retrospective analysis of 460 SARS-CoV-2-positive individuals and 976 contacts identified during an outbreak in Mede (PV), Italy, in early 2021, through routine surveillance and screening of the student population and their households (46.2% vs. 25% on average), who also caused a higher number of secondary cases (mean: 1.03 vs. 0.35), nearly threefold. The same study concludes that uncontrolled transmission of SARS-CoV-2 in schools could spread transmission to other areas and increase the burden of contact tracing. In short, there is a growing body of literature and data (see also Yan et al. 2022) showing that schools are a privileged environment for transmission and amplification of viral circulation, identifying schools as a major driver in the onward spread of infection. Therefore, it does not seem unreasonable that the mass of infections in September 2020 was strongly influenced by the reopening of schools.

The main limitation of this study is the level of aggregation used. With regional data, we were able to include air temperature as an effect modifier in the model by using the average daily temperatures of the regional capitals. This is quite reasonable since temperature is roughly constant in geographic regions that are not too large. By using data at the level of individual cities or large metropolitan areas, it would be possible to include other climate and air quality variables in the model, such as air relative humidity or $\text {PM}_{2.5}$ concentrations. From a technical point of view, it would be interesting to try to confirm and consolidate the results obtained using another purely econometric approach, such as a structural break analysis (Ahmed et al. 2017). The use of these alternative approaches will be the subject of future specific research.

Other limitations are more biological in nature and relate to the physiological characteristics and health status of individuals in the study population. Such data are not readily available, and individuals often ignore their physiological and immunological changes, which are sometimes closely related to environmental conditions (i.e., the composition and status of their microbiota) or dietary habits (e.g., healthy diet, probiotics). All these conditions may influence the susceptibility to infection and the natural history of infection in individuals (Santacroce et al. 2020, 2021), and they may also help to explain the important role of asymptomatic patients in the spread of infection.

## Data Availability

Available on the websites referenced in the article.
